# A metallic anti-biofouling surface with a hierarchical topography containing nanostructures on curved micro-riblets

**DOI:** 10.1038/s41378-021-00341-3

**Published:** 2022-01-10

**Authors:** Taekyung Kim, Sunmok Kwon, Jeehyeon Lee, Joon Sang Lee, Shinill Kang

**Affiliations:** 1 National Center for Optically-assisted high precision Mechanical Systems, Yonsei University, Seoul, 03722 Korea; 2 School of Mechanical Engineering, Yonsei University, 50 Yonsei-ro, Seodaemun-gu, Seoul, 03722 Korea

**Keywords:** Anti-biofouling, Superhydrophilic, Hierarchical topography, Metallic engineered surface, Nanoimprinting, static immersion test, field trials, Engineering, Nanoscience and technology

## Abstract

Metallic surface finishes have been used in the anti-biofouling, but it is very difficult to produce surfaces with hierarchically ordered structures. In the present study, anti-biofouling metallic surfaces with nanostructures superimposed on curved micro-riblets were produced via top-down fabrication. According to the attachment theory, these surfaces feature few attachment points for organisms, the nanostructures prevent the attachment of bacteria and algal zoospores, while the micro-riblets prohibit the settlement of macrofoulers. Anodic oxidation was performed to induce superhydrophilicity. It forms a hydration layer on the surface, which physically blocks foulant adsorption along with the anti-biofouling topography. We characterized the surfaces via scanning electron and atomic force microscopy, contact-angle measurement, and wear-resistance testing. The contact angle of the hierarchical structures was less than 1°. Laboratory settlement assays verified that bacterial attachment was dramatically reduced by the nanostructures and/or the hydration layer, attributable to superhydrophilicity. The micro-riblets prohibited the settlement of macrofoulers. Over 77 days of static immersion in the sea during summer, the metallic surface showed significantly less biofouling compared to a surface painted with an anticorrosive coating.

## Introduction

Biofouling is the unwanted accumulation of biological materials on underwater structures. Buildup of such materials causes severe malfunction of heat exchangers, ships’ hulls, and offshore plants.^1^ Biofouling increases the hydrodynamic volume and friction of submerged hulls, thus increasing power consumption.^2^ For heat exchangers, the flow rate decreases when organisms accumulate in the water channel. In the absence of anti-biofouling treatment, the channel becomes completely blocked, which leads to catastrophic failure.^3,4^ Although various chemical coatings containing biocides help to prevent marine fouling, their use has been restricted by the European Union and the International Maritime Organization because of major negative effects on marine ecosystems.^5^ The prohibition of toxic chemicals in antifouling coatings has accelerated the search for environmentally friendly alternatives. One such alternative is a fouling-release coating that modifies the surface topography and chemistry to prevent microbial settlement and attachment.

Comprehensive and methodical studies regarding engineered anti-biofouling topographies have been conducted by numerous researchers. It is commonly accepted (and experimentally proven) that an appropriate micro- or nanosurface topography modified either hydrophobically or hydrophilically reduces biofouling by various life forms via the following mechanisms.

Surface modification (hydrophobic, hydrophilic treatment) can induce antifouling. A hydrophobic surface reduces polar and hydrogen-bonding interactions with biofoulants and increases the surface-to-foulant separation distance increasing the energy required and the kinetic barriers that organisms must overcome when attaching to the surface; a hydrophilic surface strongly bind water molecules and form a hydration layer that physically blocks foulant adsorption. Yoon et al prepared (separate) superhydrophobic and superhydrophilic surfaces by annealing stainless-steel plates with carbon nanotube-polytetrafluoroethylene and titanium dioxide (TiO_2_), respectively.^6^ Bacterial growth on both surfaces was examined under static and dynamic conditions in a flow channel. Under static conditions, bacterial growth was higher on the superhydrophobic surface than on the superhydrophilic surface. The hydration layer of the superhydrophilic surface reduced bacterial growth.

If the nanostructures are smaller than the biofouler, bioadhesion and subsequent biofilm development can be halted. Seyfi et al. developed antibacterial superhydrophobic coatings based on polydimethylsiloxane (PDMS)/silver phosphate nanocomposites.^7^ Antimicrobial properties were enhanced by the low surface free energy and appropriate roughness; the surface protrusions were smaller than bacteria.

When the fouling involves larger (marine) microorganisms, topographic surfaces having a single length scale are unlikely to be as effective as conventional antifouling coatings with biocides because the organisms that engage in biofouling are diverse. Several hierarchical ordered topographies have been considered because bacteria, algae, and barnacles exhibit different settling characteristics.^8,9^ Inspired by shark skin, Schumacher et al. created hierarchical topographies, in which larger microgratings were superimposed on smaller micro-riblets. These reduced settlement of a fouling plant (Ulva) and an animal of interest (*Balanus amphitrite*). The vertical walls of the ridges, which lacked smaller micro-riblets, were favored attachment sites for Ulva zoospores. Thus, ridge walls lacking anti-biofouling patterns should be avoided.^8^

The importance of ordered structures was also emphasized in the work of Diaz et al.^10^ According to their work, the ordered structures hinder the formation of ordered aggregates of bacteria while well-defined aggregates of bacteria were formed on the randomly oriented structures. In other words, the ordered structures can pose better functionality over random structures if they are engineered for specific functions. This was also confirmed by the work of Jung et al.^11^ In their anti-icing experiment, the ordered nanostructures showed better anti-icing performance over randomly oriented nanostructures with similar dimensions to the ordered one. Thus, a hydrophobic or hydrophilic surface with a hierarchically ordered topography over the entire surface can improve anti-biofouling.

Any antifouling system must be mechanically and chemically stable in the long term; the marine environment is harsh, featuring constant exposure to salt water, sunlight, and abrasion. It has been challenging to prepare durable functional surfaces. Polymer-based antifouling coatings have been extensively studied; they are non-toxic, simple to fabricate, and well-suited to large-scale marine applications such as ships’ hulls and submerged buildings. Although polymer mechanical properties and surface chemistry can be enhanced by combination with organic, inorganic, or metallic fillers, most polymers can be vulnerable to long-term exposure to solar UV light.^12^ Such exposure causes photochemical damage near the surface; the composites then degrade. UV exposure also reduces the polymer molecular weight and thus renders it brittle, compromising the physical and mechanical properties. Apart from UV damage, some polymers such as polyethylene glycol (PEG) swell in the marine environment, compromising the mechanical properties. Also, adhesion of the coating to the metallic/composite substrate must be considered.^13^ To ensure durability, metals are very useful because of their versatile chemical, physical and mechanical properties. However, metals have seldom been used to fabricate engineered surfaces because creation of functional materials featuring ordered micro-/nanostructures is demanding in terms of both energy and time, especially if traditional processes are employed, which require a great deal of energy; metals exhibit high melting points and poor machinability.^14,15^ Also (and very importantly), metal fabrication is generally not scalable. The formation of ordered nanostructures on the tops of curved structures is even more challenging.^16^ Even though such structures can be a good candidate for the ridge-less hierarchical structure emphasized by Schumacher et al., the formation of ordered nanostructures on the tops of curved structures is challenging.

Several efforts have been made to derive hierarchical metallic surfaces via laser processing, synthesis, and etching.^17^ The hierarchical topographies fabricated by the laser-etching process were reported by numerous researchers since the method is a simple and maskless process. For example, an anti-icing aluminum alloy surface with hierarchical topography fabricated by a picosecond laser was presented.^18^ The micro-scale line structures can be uniform since it was formed by predetermined line-by-line laser irradiation path. However, the nanostructures were oriented randomly since they were formed during non-uniform vaporization of the melt and rapid solidification. The one-step hydrothermal synthesis^19^ and the HCl etching followed by H_2_O treatment^20^ were also reported for the simple fabrication of metal surfaces with micro-nano hierarchical structures. Although the above-mentioned processes are scalable, simple, it is not easy to obtain highly ordered nanostructures by the processes, which are an important factor in anti-biofouling topography.

**Fig. 1:**
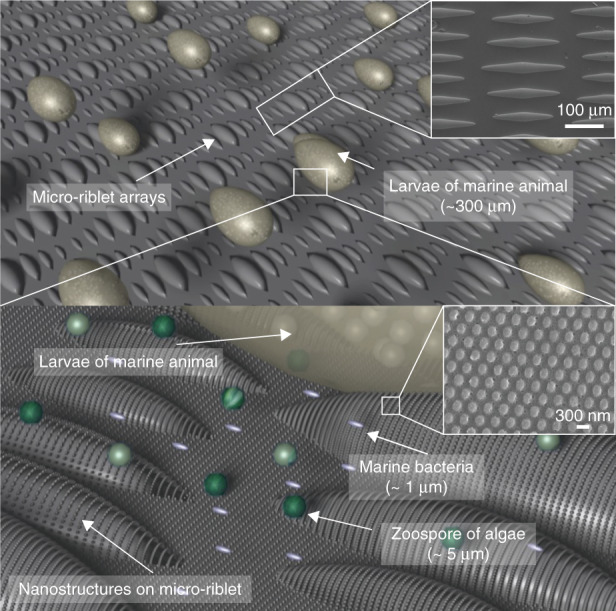
A schematic of metallic hierarchical structures with nanostructures on micro-riblets that prevent the settlement of various marine organisms including bacteria, algae, and marine animals.

In this study, we developed anti-biofouling metallic surfaces with ordered nanostructures superimposed on curved micro-riblets as shown in Fig. 1 In this study, we developed anti-biofouling metallic surfaces with nanostructures superimposed on curved micro-riblets. According to the attachment theory, the structures with smaller spacings than those of the organisms do not favor attachment because the structures make fewer contacts with the cells. In this manner, our nanostructures can reduce bacterial and algal zoospore attachment, and the micro-riblets prohibit larval fouling. We subjected the metal surface to anodic oxidation (to induce superhydrophilicity). The surface strongly binds water molecules that form a hydration layer, which physically blocks foulant adsorption. Given such strong surface hydration, it is difficult for biologicals (e.g., protein molecules, bacteria, and marine organisms) to foul the surfaces. Nanostructures were formed over the entire surface, including the micro-riblet peaks and valleys, and the bottom. Nanostructures were formed over the entire surface, including the micro-riblet peaks and valleys, and the bottom. We take a top-down approach. Laser interference lithography and conventional lithography were used for initial fabrication of the nanostructures and micro-riblets. To superimpose the nanostructures on the tops of the micro-riblets, the elastic modulus of the PDMS nanomold was modified to enhance conformal contact between the micro-riblets and the nanostructures during imprinting. Then, master hierarchical structures were replicated via pulsed reverse current (PRC) nickel electrochemical deposition; this enhances durability.^21^ Usefully, the replication process employs a reusable metallic master (Fig. S1); the process is thus practical. Once a reusable metallic master is prepared, the metallically engineered hierarchical surface can be replicated repeatedly (in one step) for as long as the passivation layer endures. Note that we eliminate the need for a seed layer; this is expensive and renders it difficult to fabricate large-area samples. Although nickel is easily oxidized in air, anodic oxidation^22^ was performed to prepare a uniform nickel oxide layer and induce superhydrophilicity.^23^ We used scanning electron microscopy (SEM), atomic force microscopy (AFM), contact-angle measurement, and wear-resistance tests to characterize the surface. The measured static contact angle of the hierarchical structures was less than 1°. Settlement assays were performed using marine bacteria (*Flavobacterium sp*.) and the larvae of a sessile animal (*Bugula neritina*). We also performed static immersion tests. After 77 days of immersion at sea, we compared our surface to a surface coated with anticorrosive paint. Both the laboratory assays and the static immersion test confirmed that our surface exhibited excellent anti-biofouling properties.

## Results and discussion

### Design of a hierarchical topography with nanostructures on curved micro-riblets

Given the complexity of the marine environment, it is difficult to identify a single antifouling topography that meets all requirements; composite antifouling coatings that combine multiple antifouling principles are required. We used two mechanisms to combat the attachment of various oceanic species. One theory that seeks to explain adhesion of cells to a substrate is the attachment point theory.^24^ When the structural spacing is larger than the sizes of the organisms, cells contact multiple potential attachment points. In such a case, cells often fit into grooves of the structures, and are thus protected from removal by water flow. On the other hand, structures with smaller spacings than those of the organisms do not favor attachment because the structures make fewer contacts with the cells. Thus, our nanostructures can reduce bacterial and algal zoospore numbers, and the micro-riblets prohibit larval fouling. Another mechanism of antifouling is the presence of a hydration layer. A superhydrophilic surface strongly binds water molecules that form a hydration layer, which physically blocks foulant adsorption. If surface hydration is strong, biologicals (e.g., protein molecules, bacteria, and marine organisms) find it difficult to displace the strongly bonded surface water molecules and thus cannot foul the surface. Our antifouling strategy features a surface with few attachment points for organisms and a hydration layer.

The dimensions of the micro-riblet structure were biomimetically determined by mimicking the skin of a shortfin mako shark (*Isurus oxyrinchus*) to simultaneously induce drag-reduction and anti-biofouling properties. The drag reduction of these structures was previously studied,^25^ which possibly aid additional antifouling because reduced drag might result in less fouling since an increased flow speed gives microorganisms less residential time to cause fouling. Considering the desired drag reduction, blade or saw-toothed structures were potential candidates but were ultimately excluded, due to their geometric instability and incompatibility with nanostructures superposition.^26^ The longitudinal and transverse lengths of the largest riblet pattern were 220 and 30 μm, and those of the smallest pattern were 130 and 25 μm. The largest gap in the micro-riblet array was about 100 µm, and the smallest was about 35 µm. The pitch of nanostructures was determined as 300 nm, as this period showed antimicrobial characteristics and low adhesion in the previous work.^27^ The length of bacteria is typically in the range of 1–3 μm and the size of larvae is in the range of 320 μm. Thus, the presented micro-riblets and nanostructures have fewer attachment points against the target organisms compared to the smooth surface. With fewer attachment points with organisms, the nanostructures can prevent the attachment of bacteria and algal zoospores, while the micro-riblets can prohibit the settlement of macrofoulers.

In terms of surface modification, we decided to induce superhydrophilicity to form a hydration layer. With strong surface hydration, it is hard for biological media (e.g., protein molecules, bacteria, marine organisms, etc.) to foul on the surfaces. Nickel oxide is hydrophilic.^23^ As the initial contact angle of bare nickel coated with nickel oxide was 56.7°, the apparent contact angle decrease as the roughness increase according to the Wenzel equation.^28^ As we superimposed the nanostructures on the micro-riblets, the hierarchical structures exhibited superhydrophilicity.

While our future works include a systematic variation of micro-and nano-patterns to investigate the effect of how variations would affect the overall results, there are several studies showing how dimensional variations in nanostructures affects the bactericidal property or anti-adhesion of bacteria.

The effect of topographical geometry on mechano-bactericidal efficacy can be found in the following two studies. In the finding of Dickson et al.,^29^ optimal nanopillar spacing was between 130 and 380 nm against *Escherichia coli* proliferation. The larger periodicity (e.g., 600 nm) exhibited less bactericidal. Wu et al. investigated the bactericidal property of various Au nanostructures including nanopillars (diameter: 50 nm), nanorings (diameter: 100–200 nm), nanonuggets (diameter: 100–200 nm). Regardless of their shapes and diameter, all three surfaces have a killing efficiency of more than 99%. However, by decreasing the height of nanostructures to 50 nm, the surface lost bactericidal property.^30^

The dimensional variation of nanostructures for anti-adhesion of bacteria, the concept we used in the study, was also reported.^31^ Three nanostructured-Si surfaces were prepared with periods of 200, 400, and 800 nm. The pillar height of all surfaces was 500 nm. The contact area fraction with bacteria for each of the nanosurfaces was 0.16, 0.02, and 0.11, respectively. Staphylococci experience weaker adhesion forces for all three nanosurfaces compared to a smooth surface, however, there was no significant difference in the adhesion force between the nanosurfaces, despite the difference in the contact area fractions. The important finding of their work lies in the bacterial detachment from a nanosurface in slight shear conditions (e.g., rinsing or dipping).

In terms of geometrical durability, instead of using sharp nanostructures for mechano-bactericidal efficacy, relatively “blunt” nanostructures with anti-adhesion of bacteria may be more suitable for use in harsh external conditions. It is expected such nanostructures experience repeated shear conditions by the wave, resulting in the detachment of marine bacteria. A systematic variation of the presented micro-and nano-patterns for optimal anti-biofouling is the subject of ongoing research.

### Fabrication of metallic hierarchical topography with nanostructures on curved micro-riblets

**Fig. 2:**
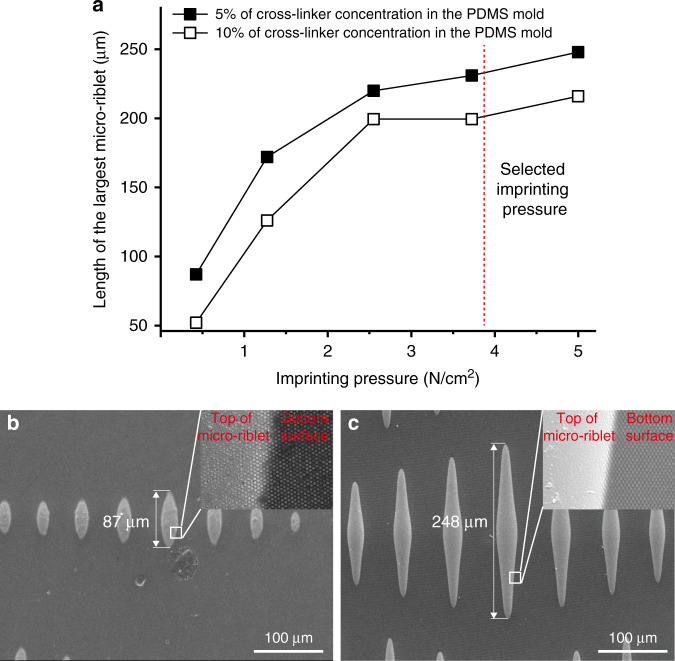
The effect of imprinting pressure and cross-linker concentration on the imprinting results. **a** Length of the longest micro-riblet as a function of imprinting pressure. A PDMS mold with a lower elastic modulus provides better conformal contact between the mold and the micro-riblets than a mold with a higher elastic modulus. Scanning electron micrographs of the imprints obtained using PDMS molds with the 5% (w/w) cross-linker. **b** 0.424 N/cm^2^ and **c** 5 N/cm^2^.

**Fig. 3:**
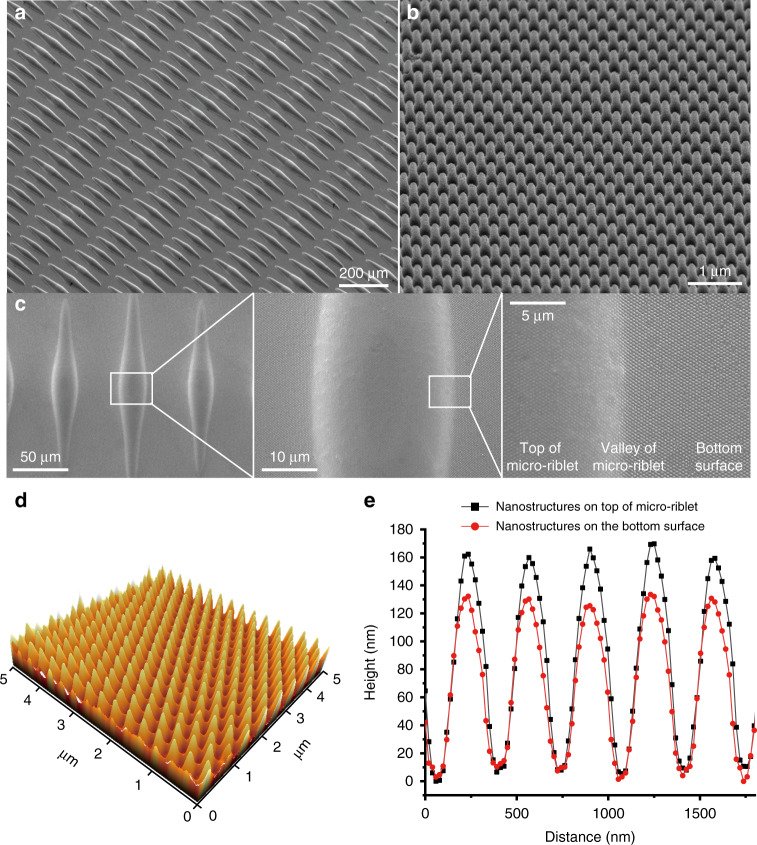
Fabrication results. Scanning electron micrographs of **a** metallic micro-riblets, **b** nanostructures from the PDMS mold, and **c** the metallic hierarchical topographies fabricated using metallic micro-riblets and the PDMS mold. In these topographies, the longitudinal and transverse lengths of the largest (center) riblet pattern were 220 and 30 μm, and those of the smallest pattern were 130 and 25 μm. **d** An atomic force microscopy 3D image of nanostructures on the bottom. **e** Surface profiles of nanostructures on the tops of the micro-riblets and on the bottoms respectively. The lattice constant and height of the bottom nanostructures were 330 and 160 nm, respectively. The nanostructures on the micro-riblets were 130 nm in height.

To ensure that nanostructures form uniformly on the tops and bottoms of curved micro-riblets, imprinting must be optimized. The key parameters to control during imprinting of nanostructures onto micro-riblets are the elastic modulus of the PDMS nanomold and the imprinting pressure. The elastic modulus depends on the concentration of the cross-linker within the PDMS.^25^ A relatively low elastic modulus provides better conformal contact between the nanomold and the micro-riblets than a high elastic modulus; the mold is less stiff than in the former case. We used a cross-linker concentration of 5% (w/w) because PDMS curing was difficult at lower concentrations. We then explored the effect of imprinting pressure on the dimensions of the riblets and nanostructures (Fig. 2a). The imprinting pressure affected micro-riblet length; for example, a relatively low pressure (0.424 N/cm^2^) was associated with a thicker residual layer and a shorter micro-riblet length (Fig. 2b). We found that an imprinting pressure of 5 N/cm^2^ deformed the nanostructures, or prohibited nanostructure formation. The resin did not remain on the tops of the micro-riblets, instead flowing to the bottoms, because the pressure was concentrated on micropattern protrusions (Fig. 2c). The optimal imprinting pressure was about 3.7 N/cm^2^, at which pressure nanostructures formed uniformly on all surfaces including the tops of the micro-riblets, the bottoms, and the interface (the micro-riblet valleys). Figure 3a shows an image of the micro-riblets that served as the substrate for imprinting. Figure 3b shows the nanostructures in the PDMS mold. Figure 3c shows the metallic hierarchical topographies fabricated using metallic micro-riblets and the PDMS mold. The nanostructures formed uniformly over the entire surface, including the tops and valleys of the micro-riblets. The nanostructure height in the PDMS nanomold was 250 nm. The heights of the nanostructures on the bottom surface and on the tops of the micro-riblets after imprinting were about 160 nm and 130 nm, respectively, as confirmed in Fig. 3d, e. The difference in height between the mold and the replica reflects volumetric shrinkage and differences in cavity filling. During UV imprinting, volumetric shrinkage upon the polymerization of UV resin is inevitable. In addition, the imprint pressure affects cavity filling, and thus the imprinted pattern volume. In general, a higher imprinting pressure ensures better cavity filling. However, we could not apply an imprinting pressure above 3.724 N/cm^2^; cavity filling was thus incomplete. In terms of the top and bottom nanostructure heights, the local imprinting pressure was higher at the tops of the micro-riblets compared to the bottoms; resin may have flowed into the bottoms. Thus, the imprinted nanostructure volume at the tops of the micro-riblets was less than that at the bottoms, explaining the lower height. Cavity filling is enhanced by higher pressure; however, a relatively low imprinting pressure should be imparted to transcribe the nanostructures to the micro-riblets during imprinting processes such as that used in this study. Enhancement of transcribability is a subject of ongoing research.

### Wettability, and mechanical and chemical robustness

Any antifouling system must be mechanically and chemically stable in the long term; the marine environment is harsh, featuring constant exposure to salt water, sunlight, and abrasion. In addition, coatings on fast (over 15 knots) water vehicles experience high levels of frictional stress. It is challenging to combine functional excellence with durability. To ensure durability, metals or metal oxides are very useful because of their versatile chemical, physical and mechanical properties. However, metals have seldom been used to fabricate engineered surfaces because creation of functional materials featuring ordered micro-/nano-structures is demanding in terms of both energy and time, especially if traditional processes are employed, which require a great deal of energy; metals exhibit high melting points and poor machinability.^14,15^ Also (and very importantly), metal fabrication is generally not scalable. Here, we fabricated a metallic surface with hierarchical structures using PRC nickel electrochemical deposition, which ensures durability.^21^ The thickness of the metallic surface is about 100 μm. Such a thin metal can be wrapped around a roll of diameter 50 mm without deforming the micro- or nano-structures, as demonstrated previously.^32^ The radius of curvature is extremely large compared to that of the flat surface of a ship or offshore platform; the radii of curvature of such structures are rather small. Thus, we believe that the thin metallic surface can be conformally integrated into curved surfaces with the exception of surfaces that are markedly curved (e.g., the front or rear of a hull). We used nickel since it has high hardness, anti-corrosion,^33^ antifouling characteristics^34^ that may make it suitable for use under harsh external conditions of the sea. Nickel is one possible candidate for the presented fabrication process; nickel alloy such as nickel–copper^35^ or nickel– phosphorus^36^ can be a promising candidate for enhanced anti-corrosion and antifouling properties.

**Fig. 4:**
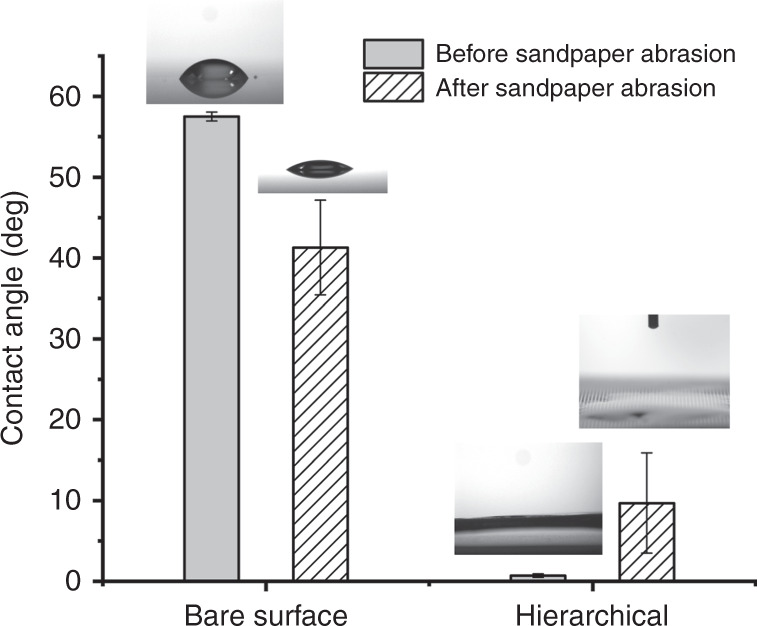
Contact-angle measurements before and after sandpaper abrasion. After placing test surfaces on 100-grit sandpaper (average particle diameter 115–162 μm), 2.58 kPa of pressure was applied using a 100-g metal weight (diameter 22 mm). Then, the surface was moved through 100 mm.

Figure 4 shows the contact-angle measurements before and after sandpaper abrasion. Nickel served as the substrate. Although nickel is readily oxidized in air, we performed anodic oxidation to create a uniform nickel oxide layer of 2–4 nm on the nickel surface.^22^ The Wenzel equation^28^ indicates that the initial contact angle (the Young contact angle) and roughness determine surface wettability. If the initial contact angle is <90°, wettability increases as roughness increases (decreasing the apparent contact angle). Nickel oxide is hydrophilic.^23^ As the initial contact angle of bare nickel coated with nickel oxide was 56.7°, the apparent contact angle decreased as the roughness increased. The contact angles of surfaces with nanostructures and micro-riblets were 26.9° and 18.1° respectively. The bare nickel surface exhibited the lowest roughness because it was replicated from a smooth silicon surface (roughness < 1 nm). The nanostructure pitch was about 300 nm. The micro-riblet gaps ranged from 35 to 100 µm. As we superimposed the nanostructures on the micro-riblets, the hierarchical structures exhibited the highest roughness values. Thus, the overall decrease in contact angle was in agreement with the work of Zhu et al.^23^ As no distinct change in hydrophilicity was evident after the finger-wipe tests, sandpaper abrasion tests were performed to further evaluate surface robustness. Our principal aim was to track changes in wettability. Thus, we did not track the mass loss before and after, which is common in the wear test. After 30 cycles of abrasion, a slight decrease in contact angle was observed for the bare surface; roughness increased. In terms of the hierarchical surface, the contact angle increased, associated with a minute deterioration in hydrophilicity, because the nanostructures on the tops of the micro-riblets had been slightly worn. When seeking durable, metallic hierarchical surfaces, the material per se needs to be considered. Relatively hard metals such as tungsten or titanium will of course wear less than softer metals such as copper or nickel. Although nickel is relatively soft, hardness can be increased in several ways, thus by varying the composition of a nickel alloy, addition of silicon carbide nanoparticles,^37^ and via PRC electrodeposition.^21^ Thicker oxide layers also enhance wear resistance and anti-corrosion, but change the nanostructural dimensions. Different corrosion characteristics between the bare metal surface and the metal hierarchical surface were evident (Fig. S4). Slight corrosion was observed over the 77-day period; the metal used was nickel. Nickel–copper or nickel–chrome alloys may be even more stable in the sea.^35^ We think that copper–nickel alloys will be of great practical benefit. Such alloys are resistant to seawater corrosion and the copper content enhances the antifouling performance. We are currently conducting relevant research including corrosion monitoring techniques.^38^

### Settlement results

**Fig. 5:**
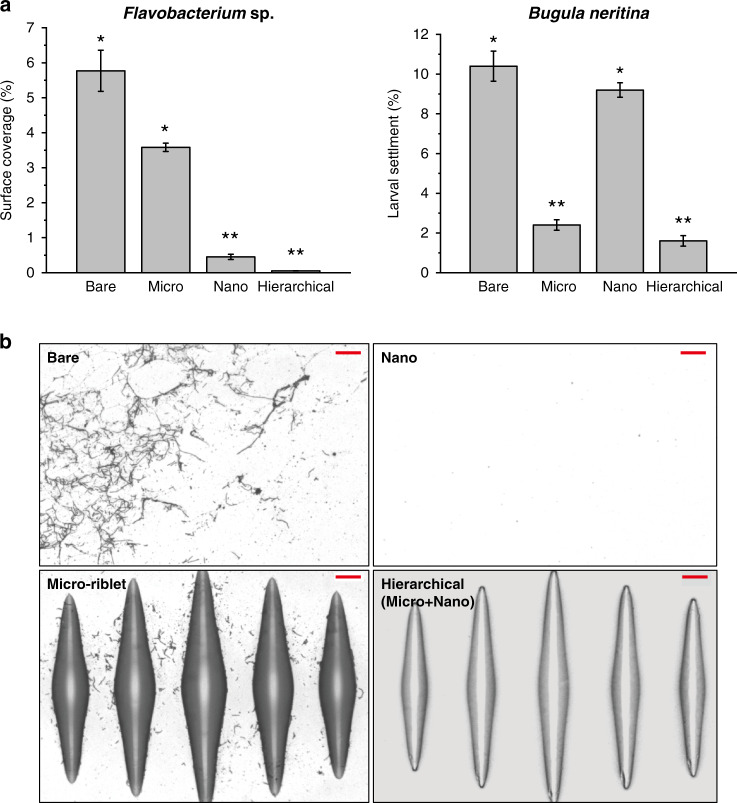
Lab settlement assays results. **a** Results of settlement assays using Flavobacterium sp. and *Bugula neritina*. *Statistically significant difference. All data are means ± SEs (*n* = 6 for Flavobacterium sp., *n* = 5 for *Bugula neritina*). **b** Optical microscopic images of samples after settlement of Flavobacterium sp. Scale bar: 10 µm. The length of a single Flavobacterium ranged from 3 to 5 μm; thus, nanostructures of pitch 350 nm prohibited bacterial attachment (as predicted by attachment point theory). As the micro-riblet gaps ranged from 35 to 100 µm (and were thus smaller than the larvae [~320 μm]), the larvae did not settle on the micro-riblets.^34^

Figure 5a shows the *Flavobacterium sp*. and *B. neritina* settlement data. *Flavobacterium sp*. grew differently depending on the type of surface [F(3,20) = 34.91, *p* < 0.001]. Both the nanostructures and the hierarchical structures exhibited significantly reduced bacterial attachment. Van der Waals and electrostatic forces, the hydration layer, and hydrophobic interactions significantly affect surface-bacterial interactions.^39^ We speculate that the Van der Waals forces between bacteria and a bare surface or micro-riblets are stronger than those between bacteria and nanostructures or hierarchical structures; sinusoidal nanostructures reduce adhesion forces.^40^ We used tryptic soy agar (TSA), pH 8.0. At this pH level, the net charge of a nickel oxide surface is slightly negative since the point of zero charge of nickel oxide is pH 7.84.^41^ *Flavobacterium* is negatively charged at this pH.^42^ Thus, a repulsive force was in play between bacteria and the nickel oxide surface. Theoretically, the repulsive force is identical for all surfaces. The Van der Waals force, the hydration layer, and hydrophobic interaction were perhaps more important; a statistically significant difference was evident between surfaces with and without nanostructures (*p* < 0.001). In terms of hydrophobic interaction, superhydrophilic nickel oxide repels the hydrophobic *Flavobacterium.*^42^ As a hydration layer was present, the superhydrophilic surface increased the surface-to-foulant distance, in turn increasing the energy required and the kinetic barriers that the organisms had to overcome to attach to the surface. Finally, the gap between nanostructures is also important. Here, the bacteria were 1–3 μm in length. Thus, nanostructures with a pitch of 350 nm repelled the bacteria, as predicted by attachment point theory.^24^ Our results are in good agreement with those of Kim et al.^27^ They stated that a topographical feature larger than a single bacterium should be avoided because bacteria are preferentially adsorbed between nanofeatures. Thus, a topographical feature size smaller than a bacterial cell reduces settlement. In addition, our design lacks ridges to which bacteria could attach.^8^ We confirmed that our nanostructures effectively prevented the attachment of marine bacteria (Fig. 5b). *B. neritina* exhibited different attachment behaviors depending on the surface type [*F*(3,8.30) = 34.91, *p* = 0.004]. Post-hoc comparisons revealed no significant difference between the smooth bare surface and the nanosurface (*p* < 0.983). Also, there was no significant difference between the hierarchical and micro-riblet surfaces (*p* < 0.882). However, a significant difference was evident between surfaces with and without micro-riblets (*p* < 0.05).

The larvae of sessile animals are motile and actively seek preferred attachment sites. *B. neritina* larvae avoid hydrophilic surfaces and seek hydrophobic surfaces.^43^ Also, topographical dimensions smaller than the larval size aid antifouling. A *B. neritina* larva is about 320 µm in length. Scardino et al. showed that the proportions of settled and metamorphosed larvae were decreased by a micrograting with a smaller period than the larval size. The largest gap in our 3D micro-riblet array was about 100 µm, and the smallest was about 35 µm. As the gap was smaller than the larval size, the micro-riblets effectively reduced larval attachment, in line with previous findings.^24^

### The static immersion test

**Fig. 6:**
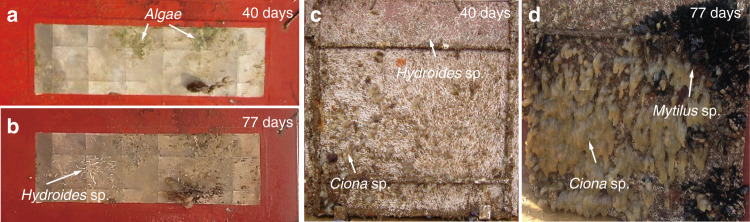
Marine static immersion test results. The engineered surface (135 × 400 mm) after immersion for **a** 40 days and **b** 77 days. The surface (600 × 600 mm) with the anticorrosive coating after immersion for **c** 40 days and **d** 77 days. The marine static immersion test was conducted during the summer at Geoje (34° 53′ 59.8″ N, 128° 37′ 06.1″ E; South Korea). The immersion depth was 1 m.

A static immersion test is essential to reveal the build-up of multiple species (bacteria, diatoms, and algae) and their interactions. The static immersion test was conducted during the summer in the sea of Geoje (34° 53′ 59.8″ N, 128° 37′ 06.1″ E; South Korea). The test surfaces remained securely bonded to the substrates after 77 days of immersion. Figure 6a, b shows images of three metal surfaces with hierarchical structures after immersion for 40 and 77 days, respectively. After 40 days of immersion, the surface remained relatively clean (a few green algae). After 40 days of immersion, the surface remained relatively clean (a few green algae). Although, after 77 days, the surface contained some *Hydroides sp*., the surface was not fully covered by algae and marine sessile animals. Certain bacterial species and strains can inhibit or stimulate algal settlement. The reason why the surface contains some degree of biofouling may be from fabrication defects or pitting corrosion, allowing bacteria or algae to attach and grow. Another possibility is that the hydration layer thickness was reduced by the shear force of water flow, allowing bacteria to attach.^6^ Figure 6c, d shows the surface painted with an anticorrosive coating after immersion for 40 and 77 days, respectively. After 40 days, the surface exhibited irreversible attachment of various marine fouling taxa including *Hydroides sp*. and *Cion sp*. After 77 days, the surface was completely covered by various hard-fouling organisms, including *Hydroides sp*., *Ciona sp*., and *Mytilus sp*.

The larvae of *Hydroides sp*. must contact a bacterial biofilm prior to settlement.^44^ Also, *Ciona sp*. is attracted by biofilms; colonization generally increases as biofilms age.^45^ We speculate that bacterial attachment was dramatically reduced by the nanostructures or the hydration layer, as confirmed in the laboratory settlement assays. In addition, we suggest that the micro-riblets played roles in preventing the attachment of macrofoulants including *Hydroides sp*. and *Ciona sp*. In previous studies, microgratings with periods of 32 and 64 µm resulted in less larval settlement than other textured surfaces.^24^ We think that our micro-riblet gaps (35 and 100 µm) are comparable to the above-mentioned values. Our metallic surfaces exhibited much less biofouling than the control surface after 77 days of immersion.

Static immersion tests do not reflect reality. Immersion tests must be performed under different flow conditions. In general, fouling decreases as the flow velocity increases.^46^ Yoon et al. investigated the effects of different flow regimes on fouling of hydrophobic and hydrophilic surfaces.^6^ Increases in the flow rate significantly decreased the numbers of *E. coli* bacteria adhering to control, hydrophobic and hydrophilic surfaces. It was also reported, loosely attached or settled bacteria could also be detached even by a gentle rinsing or dipping.^31^ The use of riblets under stationary conditions affords no obvious advantage. Benschop et al. prepared riblet-textured coatings using a commercial fouling-release paint.^46^ After immersing samples in a biofilm-culture reactor for 49 days, the samples were exposed to different flow conditions. At a maximum flow speed of 4.6 m/s, less than 1% of the biofilm remained on any tested surface. Since our hierarchical design features micro-riblets that reduce drag during turbulent flow,^25^ we believe that superhydrophilic hierarchical structures would exhibit less fouling under flow, especially turbulent flow. However, this requires further investigation because the thickness of the hydration layer can be decreased by shear, thus compromising antifouling performance.

**Fig. 7:**
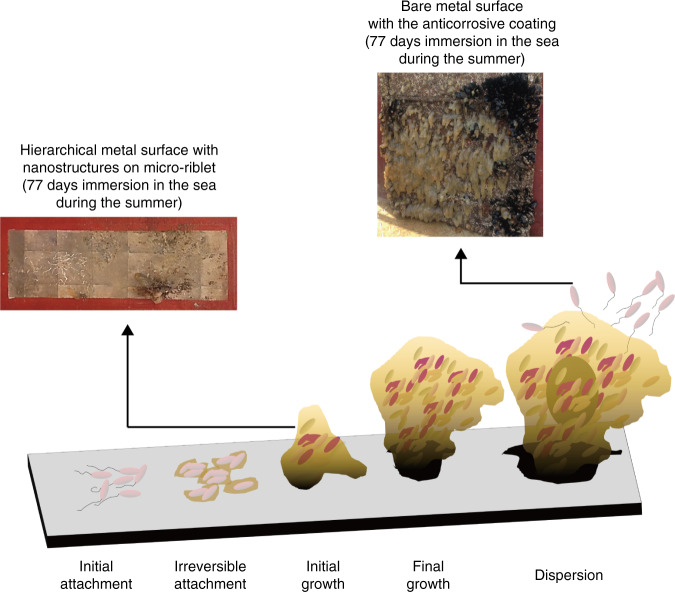
Five stages of biofilm development. It is difficult to remove a mature, complex, and structured biofilm in the dispersion stage; earlier removal is preferable.^42^

Figure 7 shows biofilm growth on the test surfaces by developmental stage.^47^ Biofilms on metals with hierarchical surfaces remained at the initial growth stage but the anticorrosive-painted surface was fully covered with a mature biofilm. It is difficult to remove a mature, complex, and structured biofilm in the dispersion stage; earlier removal is preferable. Although pitting and pinhole corrosion were found on the hierarchical metal surface after mechanical cleaning, the rest of the surface was intact (Fig. S4). However, noticeable defects were evident in the bare metal surface coated with the anticorrosive; mechanical cleaning removed both irreversibly attached marine organisms and the coating. Although the SPC paint coating the areas that did not bear the metallic surface was very effective in terms of anti-biofouling action, the paint is toxic. It is also worth mentioning that the test surface remains secure bonding with the substrate after the immersion tests.

### Comparison with other antifouling technologies

The prohibition of toxic metal compound use in antifouling coatings has accelerated the search for environmentally friendly alternatives. One approach is the development of non-toxic environmentally friendly antifoulants that replace metal compounds; thus, the use of natural antifouling substances (e.g., materials from *Chondrus crispus*^48^ and *Nerium oleander L.*^49^). Such natural extracts are typically blended into paints that easily coat even complex surfaces (this is commercially important). Also, the materials can be coated on top of commercial anticorrosive paint, thus enhancing corrosion resistance and facilitating maintenance. However, although many biological antifoulants have been identified, in-depth research on the extraction methods and efficiency are required. In addition, before these promising extracts are widely used, their degradability and toxicity to non-target organisms need to be investigated.

Another approach (which we employ) is the use of biomimetic micro- or nanostructured surfaces. A biomimetic antifouling coating contains no antifoulant and will not release compounds into the sea. It is thus eco-friendly. However, fabrication is often complicated and not scalable. These concerns must be addressed. Given the diversity of marine organisms, a single antifouling mechanism may not be adequate; composite coatings that combine various antifouling principles are required. The biomimetic approach could be enhanced by combining chemical and physical antifouling attributes.

To address the issues, polymer-based antifouling coatings have been extensively studied; they are non-toxic, simple to fabricate, and well-suited to large-scale marine applications such as ships’ hulls and submerged buildings. PDMS is a promising base material for non-toxic antifouling coatings; the methyl groups endow the surface with hydrophobicity and low energy. PDMS has a low elastic modulus; this reduces the adhesive force of marine organisms.^13^ Polyurethane-based coatings have also been studied because their mechanical properties are better than those of PDMS. Although polymer mechanical properties and surface chemistry can be modified by combination with organic, inorganic, or metallic fillers, most polymers are degraded by long-term exposure to solar UV light.^12^ Such exposure causes photochemical damage near the surface; the composites then degrade. UV exposure also reduces the polymer molecular weight and thus renders it brittle, compromising the physical and mechanical properties. Although PDMS exhibits good resistance to UV light, such light changes the surface wettability, compromising the engineered functionality.^50^ Apart from UV damage, some polymers such as PEG swell in the marine environment, compromising the mechanical properties.^13^

As one possible method to overcome this limitation, we present a method for scalable fabrication of a superhydrophilic, anti-biofouling, engineered metallic surface with ordered nanostructures on curved micro-riblets. Note that we eliminated any need for a seed layer, which is expensive to deposit and renders it difficult to fabricate large-area samples. We believe that our work opens a path toward scalable fabrication of antifouling metal surfaces.

## Conclusions

It is very difficult to produce metal surfaces with hierarchically ordered structures, although these would find many industrial applications, including anti-biofouling. Also, metal fabrication of ordered structures is generally not scalable. Here, we fabricate a superhydrophilic, anti-biofouling, engineered metallic surface featuring ordered nanostructures on curved micro-riblets. Fabrication featured replication employing a reusable metallic master; the process is practical. Roll-to-roll-based replication or direct metal imprinting is also possible. We took a top-down approach that yields hierarchically structured metal surfaces that are both stable and uniform, and thus yield statistically meaningful data. To induce superhydrophilicity, the metal replica was subjected to anodic oxidation. The measured contact angles of the hierarchical structures were <1°. A slight increase in contact angle was observed after mechanical abrasion using sandpaper, but the superhydrophilicity remained. Laboratory settlement assays verified that bacterial attachment was dramatically reduced by the nanostructures and the hydration layer. Micro-riblets prevented settlement of the macrofouler *B. neritina*. Over 77 days of static immersion in the sea during summer, the metallic surface exhibited significantly less biofouling compared to a surface painted with an anticorrosive.

We suggest that our work opens up a path toward scalable fabrication of eco-friendly, antifouling metal surfaces with ordered micro- and nanohierarchical structures that will find applications in the marine and medical industries. We believe that various combinations of useful anti-biofouling topographies could exploit our fabrication method. Superimposition of various nanostructures on top of micro-riblets, and the use of alloys, are subjects of ongoing research.

## Materials and methods

### Fabrication of superhydrophilic metallic hierarchical structures

**Fig. 8:**
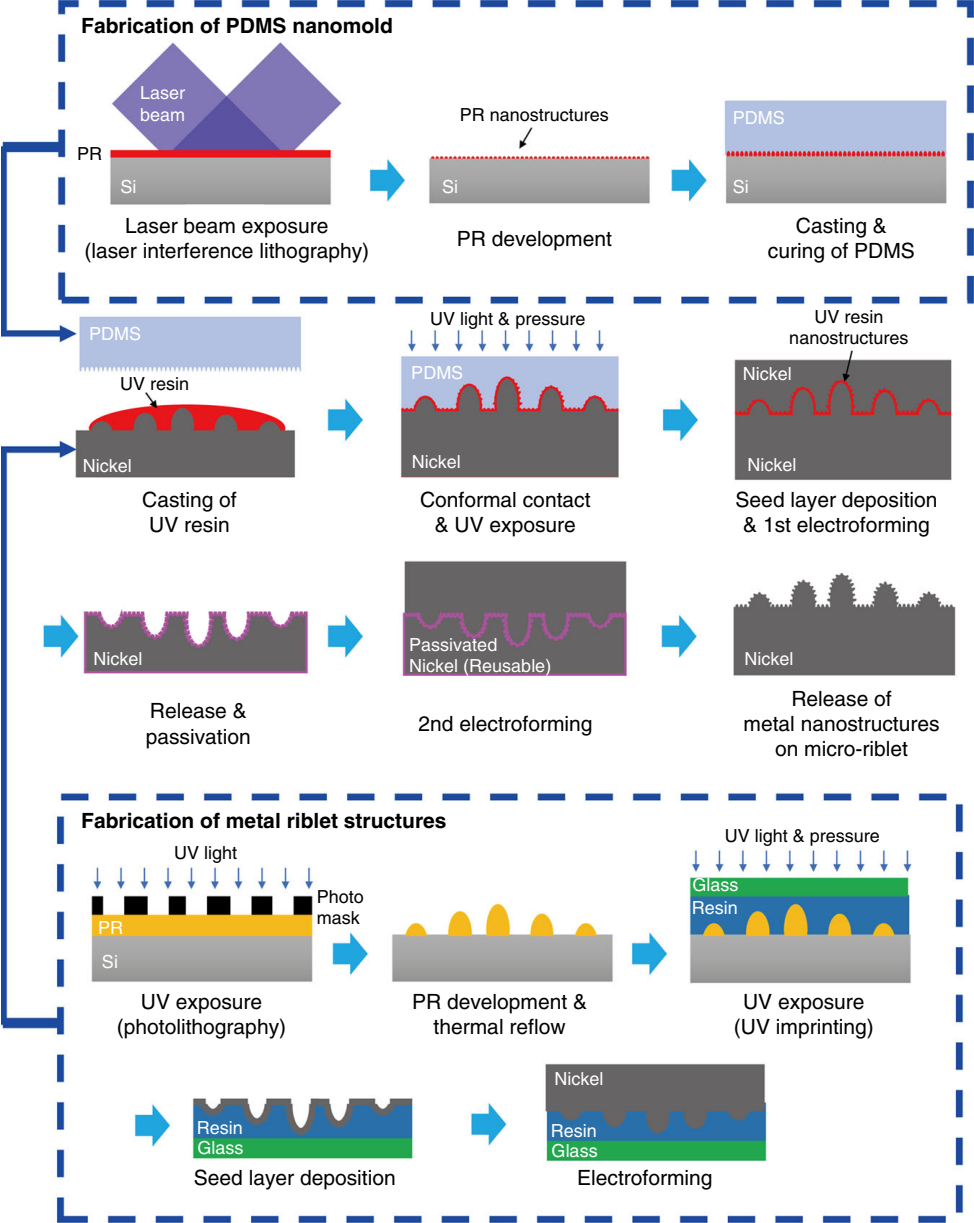
Fabrication of an anti-biofouling, metallic engineered surface.

Figure 8 shows a schematic of the fabrication process. To fabricate metallic micro-riblets, a photoresist (PR) master was created via photolithography followed by thermal reflow. Negative polymer structures were replicated from the PR master via nanoimprinting. After deposition of a conductive layer on the tops of the replicated structures, PRC electroforming was used to fabricate the metallic micro-riblets. A PDMS mold with sinusoidal nanostructures was replicated from a PR master fabricated via laser interference lithography. After polymeric nanostructures had been nano-imprinted on the tops of the metallic micro-riblets using the PDMS mold, a metallic master with a hierarchical topography was prepared via PRC electroforming. The metallic master was then subjected to additional PRC electroforming to fabricate metallic hierarchical structures with nanostructures on micro-riblets. During the interval between the consecutive electroforming processes, an oxide layer a few nanometers in thickness (a passivation layer) was created on the negative metallic replica to prevent metal inter-diffusion between the metallic master and the metal replica. To induce superhydrophilicity, the metal replica was subjected to anodic oxidation. The detailed fabrication of metallic hierarchical structures was described in our previous study.^25^

### Surface characterization

The static contact angle was measured by a contact-angle analyzer (Phoenix 300, SEO) using the sessile drop method. A 6-µL droplet of deionized water was placed on the surface at room temperature. Three measurements were taken at the same location, and the data were averaged. SEM micrographs were taken using a Field Emission platform (JEOL-7001F, Japan) at an acceleration voltage of 5 kV. Surface nanostructural profiles were assessed with the aid of AFM (Park Systems NX10, Korea). Surface robustness was assessed by the finger-wipe and sandpaper abrasion tests. Each surface was pressed and wiped 20 times with a bare finger. To assess sandpaper abrasion, after placing the test surfaces on 100-grit sandpaper (average particle diameter 115–162 μm), 2.58 kPa of pressure was applied using a 100-g metal weight (diameter 22 mm). Then, the surface was moved through 100 mm.

### Settlement assays with *Flavobacterium sp*

*Flavobacteria* are common in marine environments, normally in slime, and affect the settlement of marine organisms including other bacteria, algae, and diatoms. Flavobacteria significantly increase settlement of *Ulva linz*a. *U. linza* is a very common model to investigate settlement and adhesion of algae.^51^ *Flavobacterium* sp. was obtained from the Korean Collection for Type Cultures (KCTC, Korea) and cultured on TSA. After 48 h of cultivation, a *Flavobacterium sp*. suspension was diluted to an optical density of 0.5 at 600 nm using an ultraviolet-visible spectrophotometer (U-3010; Hitachi, Japan). To initiate adhesion, 50 mL amounts of the diluted suspension were placed in Petri dishes containing test samples, and the dishes shaken at 50 rpm at 30 °C for 3 h to allow bacterial adhesion.^52^ Subsequently, planktonic (unbound) or loosely attached cells were removed via repetitive rinsing with phosphate-buffered saline. The adhered bacteria were stained with a crystal violet solution for 1 min, washed in distilled water; stained in an iodine-potassium iodide solution for 1 min, washed again and dried for 20 min at 50 °C. As described in,^53^ the areas covered by bacteria were calculated after converting Gram-stained images to binary images.

### Settlement assays with *Bugula neritina*

*B. neritina* is of major concern in terms of biofouling; this is a globally common marine bryozoan.^54^ These larvae frequently attach to boat hulls; the species is regarded as one of the most widespread fouling bryozoans. Adult *B. neritina* were provided by the Marine Bryozoans Resources Bank of Korea (MBRBK; Woosuk University, Korea). Colonies of *B. neritina* were placed in aerated seawater in the dark for 24 h. After 15 min of exposure to light (to allow for spawning), approximately 50 larvae were pipetted into Petri dishes containing test samples. The dishes were covered and kept in the dark for 24 h to allow for larval settlement, which was enough for the larvae to metamorphose. After the settlement assay, the fully metamorphosed larvae (Fig. S3) were only considered as attached to the surface. The numbers of metamorphosed larvae in each sample were counted under an optical microscope (Olympus STM6-LM, Japan) and the percentage settlement was quantified.

### Statistical analysis

Statistical analysis yields meaningful experimental data, especially when dealing with living organisms (as in most antifouling or antimicrobial research). To evaluate bacterial settlement, six fields of view from each of six replicates were examined. The images were converted to binary format using ImageJ software (NIH, USA) and the bacterial surface coverage was determined by the ratio of the black area (indicating bacterial attachment) to the total area. The bacterial surface coverage was calculated as the average of those of the six images. Statistical differences were evaluated by one-way analysis of variance (ANOVA) followed by Tukey’s HSD post-hoc test for multiple comparisons. The settled *B. neritina* larvae were counted on five replicate surfaces. The Welch ANOVA was used due to heterogeneity of variance. Games-Howell post-hoc analysis was performed for multiple comparisons. All data were arcsine-transformed prior to statistical analysis. This improved the normality and reduced variance. All data are presented as mean ± standard error. The level of statistical significance was set to 5%. All statistical computations were performed using the Minitab 1 (ver.19; Minitab Inc, State College, USA) statistical software package.

### Static immersion test in the sea

The surface used in the immersion test was 135 mm in width, 400 mm in height, and 100 μm in thickness. We performed step-and-repeat imprinting and electroforming using a 70 × 70-mm metal stamp (Fig. S1) with nanostructures on micro-riblets to prepare the surfaces. An anticorrosive paint (KNA 340) was applied to both sides of a carbon steel panel (600 × 600 mm). Next, an engineered surface was attached to one side of the panel using epoxy resin. The regions without metal surfaces were painted with a self-polishing copolymer (SPC) antifouling paint. As the control, we used carbon steel panels coated with the anticorrosive paint. After preparation, both panels were fixed to a raft and immersed in the sea at Geoje (34° 53′ 59.8″ N, 128° 37′ 06.1″ E; South Korea) for 77 days in summer. The immersion depth was 1 m.

## Supplementary information

Supplementary Materials
