# Supplementary material for: A metallic anti-biofouling surface with a hierarchical topography containing nanostructures on curved micro-riblets
Source: Microsyst Nanoeng. 2022 Jan 10;8:6. doi: 10.1038/s41378-021-00341-3 (PMC8743286; doi:10.1038/s41378-021-00341-3)
Supplement: Supplementary file 1 — Supplementary Materials [file 41378_2021_341_MOESM1_ESM.docx]

Supplementary Materials

Title: A metallic anti-biofouling surface with hierarchical topography containing nanostructures on curved micro-riblets

Taekyung Kim^b^, Sunmok Kwon^a,b^, Jeehyeon Lee^a,b^, Joon Sang Lee^a,b^, Shinill Kang^a, b, *^

^a^School of Mechanical Engineering, Yonsei University, 50 Yonsei-ro, Seodaemun-gu, Seoul, 03722, Korea

^b^National Center for Optically-assisted high precision Mechanical Systems, Yonsei University, Seoul, 03722, Korea

*Corresponding authors: [snlkang@yonsei.ac.kr](mailto:snlkang@yonsei.ac.kr)

**1. Reusable negative metallic master**

When fabricating the metallic engineered surfaces, we included pulse reverse current (PRC) electrodeposition “atom by atom” on the master. Thus, nanoscale replication is possible; the dimensions of the mold and the replica do not differ [1].

Using a negative metallic mold (Figure S1a), electrochemical deposition yielded several positive replicas. Figure S1b and S1c show SEM images of the micro-riblets and nanostructures respectively. To combine the replicas into one body, they were laser-welded as shown in Figure S1d. Figure S1e and S1f show SEM images of the micro-riblets and nanostructures respectively. Figure S1g compares the surface profiles of the master and replica micro-riblets and nanostructures. Table S1 shows the comparison of dimensions between master and replica. The profiles of micro-riblets and nanostructures were measured by confocal microscopy and AFM, respectively.

During metal-to-metal electrodeposition, an oxide layer a few nanometers in thickness (a passivation layer) is required to prevent metal inter-diffusion between the negative metallic master and the metallic engineered surfaces. Although a passivation layer on a negative metallic stamp may deteriorate after several metal-to-metal electrodeposition steps, the layer can be regenerated either electrochemically or chemically [2].


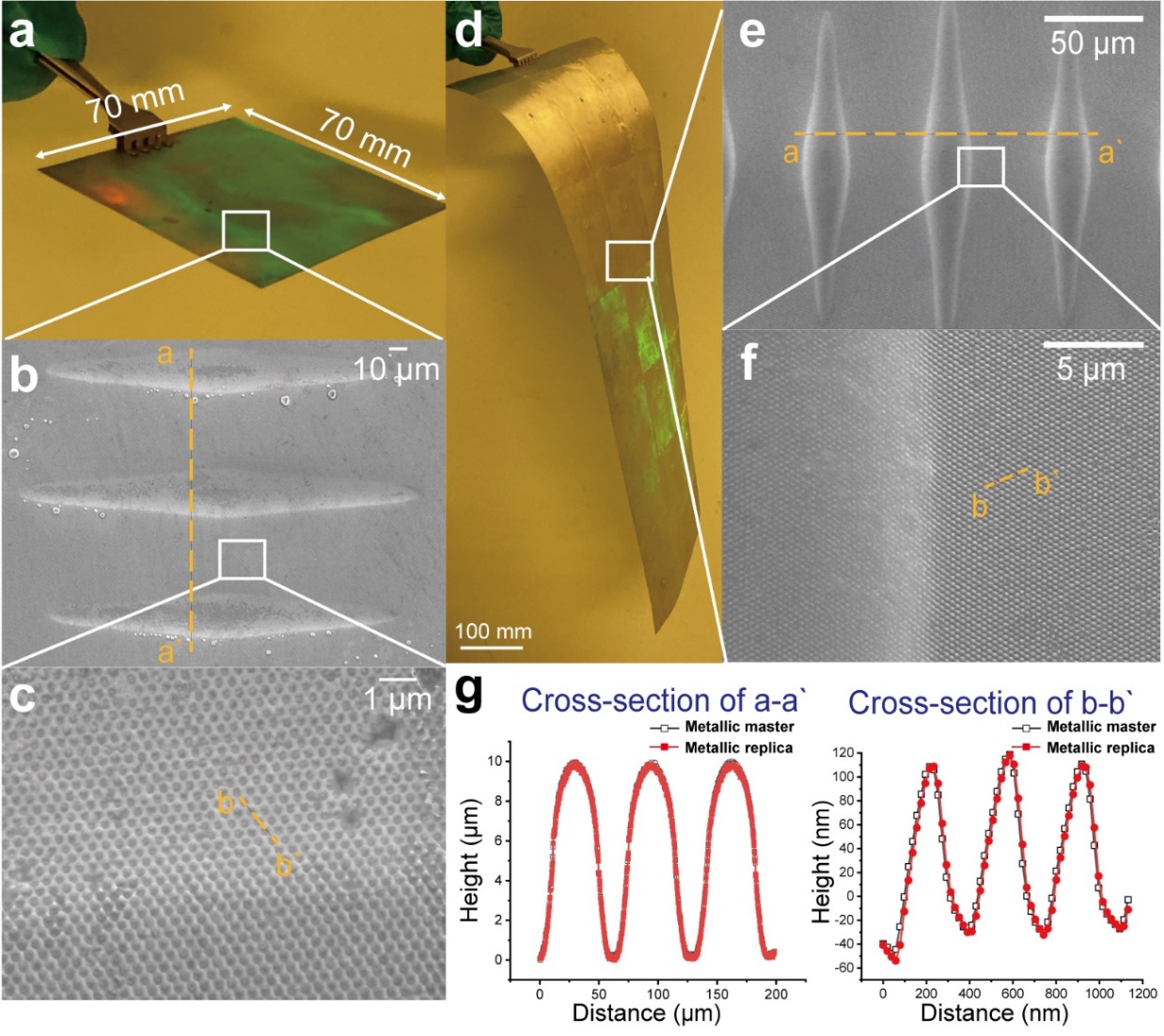


**Fig. S1.** A photograph of the metallic mold used for electrochemical deposition. SEM images of the mold show (b) the negative shapes of the micro-riblets and (c) the negative shapes of the nanostructures. (d) A photograph of the laser-welded metallic surfaces. SEM images of the metallic surface (e) show the positive shapes of the micro-riblets and (f) the positive shapes of the nanostructures. (g) A comparison of the surface profiles of the master and replica micro-riblets and nanostructures.

Table S1. Comparisons between the master and the replica.

|  | Micro-riblets | | | | | Discrepancy (%) | Nanostructures | | | | Discrepancy (%) | |
| --- | --- | --- | --- | --- | --- | --- | --- | --- | --- | --- | --- | --- |
|  | Master | | Replica | | |  | Master | | | Replica |  |  |
| Height | | 10.01 µm | | 9.93 µm | 0.78 % | | | 150.09 nm | 149.12 nm | | | 0.64 % |
| Base width | | 29.63µm | | 29.59 µm | 0.13% | | | 351.60 nm | 351.50 nm | | | 0.03 % |

**2. Pulse-reverse current (PRC) electroforming**

Figure S2a shows a photograph of the PRC electroforming system (Technotrans AG, Germany). Figure S2b shows a schematic diagram of PRC, where T_off_ is the anodic time, T_on_ the cathodic time, and I_average_ the average current density. PRC electroforming features cycles of metal deposition and dissolution controlled by the current waveform. When a positive pulse current (I_cathode_) is applied, a cathodic reaction occurs and metal is deposited on the cathode surface; a negative pulse current (I_node_) causes the reverse effect (the deposited metal is dissolved by the anodic reaction). Thus, metal replication via PRC electroforming is associated with a smaller grain size than is metal replicated via direct current electroforming. As smaller crystals fill cavities in deposited metal more densely than do larger crystals, the replicated metal exhibits fidelity and durability. Table S2 summarizes the PRC electroforming conditions. We used a duty cycle of 0.7, a frequency of 300 Hz, and an average current density of 120 mA/cm^2^.

**Fig. S2.** (a) Photograph of PRC electroforming system (Technotrans AG, Germany) (b) Schematic diagram of PRC, where T_off_ is the anodic time, T_on_ is the cathodic time, and I_average_ is the average current density

Table S2. Processing conditions for PRC electroforming process

| Process temperature | 52–54°C |
| --- | --- |
| Potential of hydrogen | 3.5–3.6 |
| Total volume of electrolyte | 240L |
| Flow rate of electrolyte | 64L/min |
| Concentration of Ni(NH_2_SO_3_)_2_·4H_2_O | 345–355g/L |
| Concentration of NiCl_2_·6H_2_O | 5g/L |
| Concentration of H_2_BO_3_ | 35g/L |

**3. Developmental stages of Bugula neritina**

In terms of larval species selection, *Bugula neritina* is of major concern in terms of biofouling; this is a globally common marine bryozoan. The larvae frequently attach to boat hulls; the species is one of the most widespread fouling bryozoans [3]. Two articles have explored the settlement duration required for larval attachment. Li et al. allowed the larvae to settle for 4 h [4]; Price et al. recorded the number of attached larvae at 2, 4, 6, 8, 24, and 48 h following the start of treatment [3]. Li et al. found that larvae settled and metamorphosed after only 4 h; however, we allowed 24 h of settling. Figure S3a and S3b show the control metal surface before and after larval attachment respectively. The numbers of metamorphosed larvae in each sample were counted.


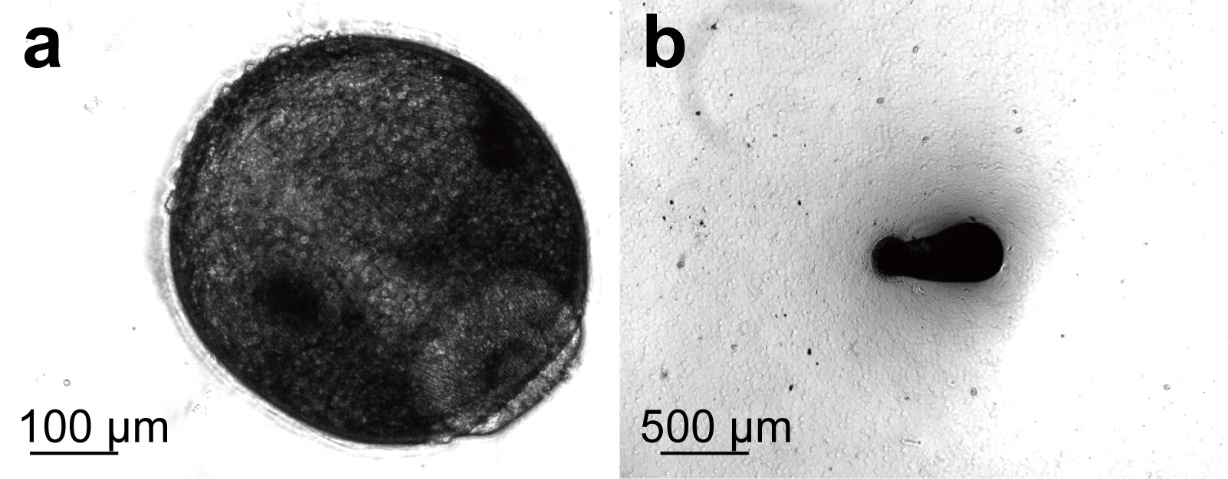


**Fig. S3.** Developmental stages of Bugula neritina (a) swimming larvae immediately released from adult (b) complete larval attachment to a control metal surface after 24 h of settlement assays

**4. Mechanical cleaning after sea immersion test**

Figure S4 shows the 77-day marine static-immersion test results before and after mechanical cleaning. Although pitting corrosion was observed in certain regions of the hierarchical metal surface after cleaning, the other surface regions remained intact. We plan to change the base material from nickel to a nickel-alloy. Copper-nickel alloys strongly resist seawater corrosion and copper enhances anti-fouling [5]. We are currently conducting relevant research.

Biofilms on metals with hierarchical surfaces remained at the initial growth stage but the bare metal surface was fully covered with a mature biofilm. It is difficult to remove a mature, complex, and structured biofilm in the dispersion stage; earlier removal is preferable.

We have added the figures in the supplementary material.


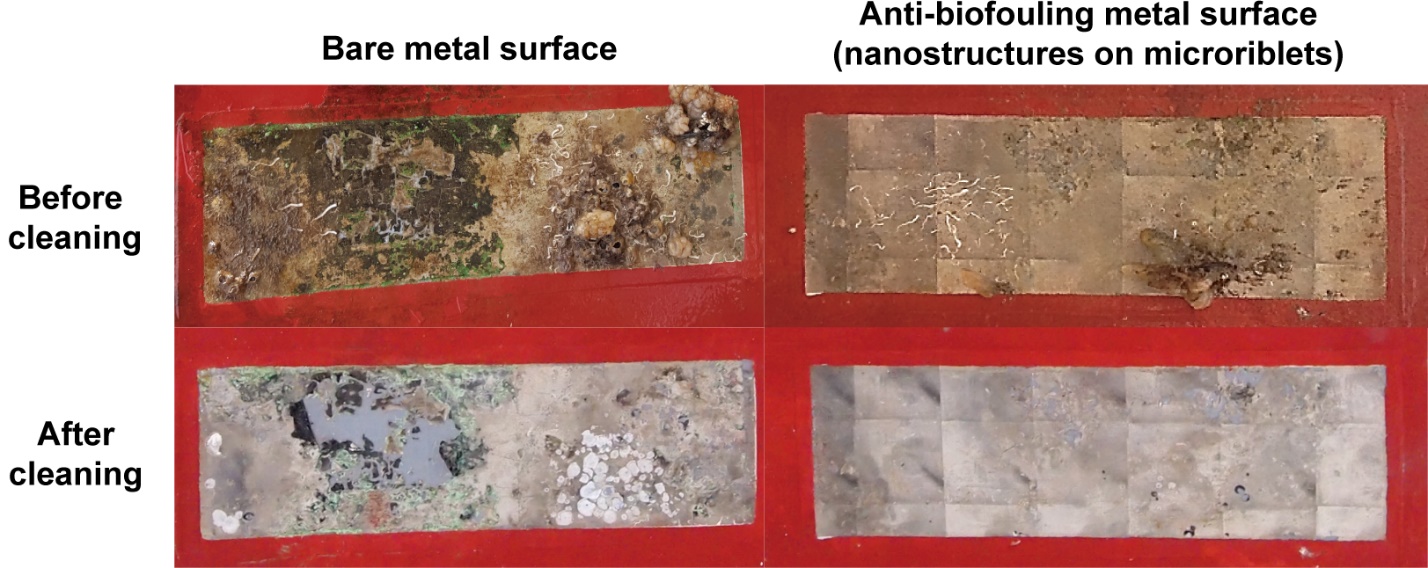


**Fig. S4.** The 77-day marine static-immersion test results before and after mechanical cleaning. Although pitting and pinhole corrosion was found on the hierarchical metal surface after mechanical cleaning, the rest of the surface was intact. However, noticeable defects were evident in the bare metal surface.

[1] J. Kim, J. Han, T. Kim, S. Kang, Fabrication of a metallic roll stamp with low internal stress and high hardness for large area display applications by a pulse reverse current electroforming process, J. Micromech. Microeng. 24 (2014) 125004. https://doi.org/10.1088/0960-1317/24/12/125004.

[2] E.-S. Hwang, J.-W. Park, J.-G. Kim, Y. Cho, K.-M. Yeo, J.-W. Seo, H. Kim, S. Lee, Micro Pattern Roll Mold for Large Area Display by Electroforming and Wrapping Method, Jpn. J. Appl. Phys. 48 (2009) 050211. <https://doi.org/10.1143/JJAP.48.050211>.

[3] H.L. Price, N.V. Gohad, A.S. Mount, D.E. Wendt, Investigation of larval settlement pathways in the marine bryozoan, Bugula neritina, Journal of Experimental Marine Biology and Ecology. 486 (2017) 69–76. https://doi.org/10.1016/j.jembe.2016.09.017.

[4] H.-X. Li, B. Orihuela, M. Zhu, D. Rittschof, Recyclable plastics as substrata for settlement and growth of bryozoans Bugula neritina and barnacles Amphibalanus amphitrite, Environmental Pollution. 218 (2016) 973–980. https://doi.org/10.1016/j.envpol.2016.08.047.

[5] Kh. Rahmani, R. Jadidian, S. Haghtalab, Evaluation of inhibitors and biocides on the corrosion, scaling and biofouling control of carbon steel and copper–nickel alloys in a power plant cooling water system, Desalination. 393 (2016) 174–185. https://doi.org/10.1016/j.desal.2015.07.026.
